# Recreational noise induced hearing loss: South African undergraduate students’ perspectives

**DOI:** 10.3389/fpubh.2025.1534731

**Published:** 2025-03-18

**Authors:** Katijah Khoza-Shangase, Khothatso Mokhethi

**Affiliations:** Department of Audiology, School of Human and Community Development, University of the Witwatersrand, Johannesburg, South Africa

**Keywords:** recreational noise-induced hearing loss, earphones, undergraduate students, South Africa, hearing health awareness, personal listening devices

## Abstract

**Background:**

Recreational noise-induced hearing loss (NIHL) is an increasing public health concern among young adults who frequently use personal listening devices (PLDs) at high volumes for extended periods. Despite this, awareness of NIHL risks remains low, particularly among university students in South Africa.

**Objective:**

This study aimed to assess undergraduate students’ awareness of recreational NIHL, examining their earphone use habits, volume preferences, preventive behaviors, and associations between demographic variables and NIHL awareness.

**Methods:**

A cross-sectional survey was conducted with 154 undergraduate students at a large urban South African university. Quantitative data on listening habits, NIHL awareness, and preventive behaviors were collected and analysed using descriptive including thematic analysis and inferential statistics such as Chi-square tests to examine associations between variables.

**Results:**

Most participants (67.5%) reported daily earphone use, often at moderate (48.1%) or high (33.8%) volumes. Awareness of NIHL was low, with only 9.7% of students feeling very informed. Inferential analysis revealed significant associations between NIHL awareness and age (*χ*^2^ = 12.67, *p* < 0.05), as well as year of study (*χ*^2^ = 10.89, *p* < 0.05), with older students and those in upper academic years (third year or beyond) showing greater awareness. Preventive behaviors were inconsistent; 46.1% of students reported lowering volume, while 13.0% took no preventive measures. Further analysis revealed that students who preferred high volumes were more likely to adopt preventive measures, while those who preferred low volumes often took no action, perceiving their existing habits as safe. Thematic analysis identified concerns about hearing health, barriers to safe listening, influence of social norms, and misconceptions about ear health.

**Conclusion:**

The findings highlight a high prevalence of potentially unsafe listening behaviors and low NIHL awareness among South African university students. Recommendations include university-based hearing health programs, leveraging social media for outreach, integrating education into first-year curricula, and promoting affordable protective options, and social media campaigns targeting safe listening practices. These measures could help foster safer listening habits and reduce NIHL risk within this vulnerable population.

## Introduction

Noise-induced hearing loss (NIHL) has become one of the most common, yet preventable, health concerns worldwide, affecting millions of individuals and contributing to long-term, irreversible hearing impairments (1). Recreational NIHL specifically refers to hearing loss resulting from exposure to high sound levels in leisure settings, including the use of personal listening devices (PLDs) like earphones and headphones (2). As these devices become ubiquitous, especially among young people, the risk of NIHL has increased significantly, with studies indicating that up to 1 billion young individuals worldwide are at risk due to unsafe listening practices with PLDs (3). The WHO reports that globally, these unsafe listening practices are driven largely by prolonged and high-volume use of PLDs (4). With increased urbanization, access to advanced technology, and the normalization of portable audio devices, the potential for recreational NIHL among youth has intensified.

South Africa presents a unique context within this global concern, as it balances rapid technological adoption with persistent socioeconomic and healthcare challenges. The widespread use of smartphones, estimated to be at 26.3 million users, has made PLDs accessible to a broader demographic, including South Africa’s large youth population (5). For many young adults, especially university students, earphones are integrated into daily routines—whether for commuting, studying, exercising, or relaxing. However, research within South Africa has shown that knowledge and awareness of the risks associated with NIHL remain limited among young adults (6, 7). Unlike high-income countries (HICs), where public health interventions and educational programs on NIHL are more established (4, 8), South Africa faces structural challenges in implementing widespread hearing health education (9, 10). Consequently, while South African youth may engage in similar listening behaviors to their global counterparts, they are at heightened risk of unknowingly adopting unsafe listening practices.

Noise-induced hearing loss is a type of sensorineural hearing loss that arises from prolonged exposure to excessive noise, leading to permanent damage to the delicate sensory hair cells within the cochlea of the inner ear (11). This type of hearing impairment is marked by the absence of regenerative capacity in the affected cells, meaning that once hearing is lost, it cannot be restored. NIHL can arise from both occupational and recreational exposure to noise (10). While occupational NIHL, caused by prolonged noise exposure in work environments, has long been recognized and legislatively addressed (12, 13), recreational NIHL is increasingly relevant in the context of PLDs (14–16). High-intensity sound levels—often exceeding the globally recommended limits of 85 dBA—can induce temporary or permanent hearing threshold shifts, leading to issues such as tinnitus and reduced speech intelligibility (17, 18). Studies have shown that prolonged exposure to sound levels above 85 dBA, a commonly used occupational exposure limit globally, can result in permanent hearing damage. This threshold is designed to protect most individuals from significant hearing loss over an 8-h daily exposure period; however, some organizations recommend lower limits, such as 80 dBA or 70 dBA, for enhanced protection. As young people in South Africa and globally increasingly use PLDs at high volumes, the associated risk of NIHL becomes more pronounced.

For undergraduate students, particularly those studying in high-noise urban environments, PLDs often serve as a mechanism to create a personal auditory space (19, 20). This practice has seen consistent growth, yet it is often coupled with potentially unsafe listening behaviors, such as extended listening durations and high-volume settings (19, 21–23). Studies across various countries indicate that young people frequently exceed recommended listening thresholds, such as 85 dBA, and disregard device warnings about high volume exposure (24–26). However, recreational noise exposures, which are often more intense or prolonged, may hinder auditory recovery and contribute to permanent hearing damage even at lower exposure levels The lack of adequate educational programs targeting safe listening practices exacerbates this issue. Studies have shown that a large percentage of students reported no awareness of recreational NIHL risks, with many unsure of the long-term consequences of high-volume listening (7, 23). In South Africa, the scarcity of research on earphone use among university students further limits the potential for targeted interventions that could address these behaviors and improve awareness.

Moreover, South Africa faces unique challenges in addressing this public health concern. While global efforts to prevent NIHL often rely on technological interventions and awareness campaigns, South Africa’s educational and public health sectors may lack the resources to deploy similar strategies comprehensively. South Africa faces structural challenges when compared to HICs, partly due to limited public health funding. For example, South Africa’s public health expenditure accounts for approximately 8.3% of GDP (4), compared to an average of 12% in many developed nations. These funding disparities can hinder the implementation of widespread hearing health education and access to advanced technological interventions. Studies suggest that students’ awareness of safe listening practices is limited, partly due to the absence of consistent messaging within the healthcare and educational frameworks (7, 27, 28). With a youth-dominated population and increasing access to advanced technologies, the risk of recreational NIHL could rise without timely interventions.

Recognizing the importance of context-specific research, this study aims to investigate undergraduate students’ awareness and understanding of recreational NIHL in South Africa, with particular focus on their earphone use habits, volume preferences, preventive behaviors, and associations between demographic variables and NIHL awareness. By exploring students’ awareness of the risks associated with recreational NIHL, this study seeks to inform targeted public health strategies that are not only preventative but also culturally and economically feasible within the South African context. Through such initiatives, the country can take critical steps toward mitigating the risk of NIHL associated with the uncontrolled use of PLDs and fostering healthier listening behaviors among its youth population.

## Methodology

### Research design

This study employed a cross-sectional, non-experimental, descriptive phenomenological mixed-methods design using a quantitative survey method (29, 30), to examine the awareness, attitudes, and behaviors regarding recreational NIHL among undergraduate students. A cross-sectional design was selected to capture a snapshot of students’ knowledge and behaviors regarding earphone use and NIHL risk, whereas the mixed approach allowed for the identification and quantification of patterns and trends within the sample, while establishing relationships between variables.

### Study setting and population

The study was conducted among undergraduate students at a large urban university in Johannesburg, South Africa. The institution, located in Gauteng Province, has a significant proportion of South Africa’s youth enrolled, with a current undergraduate population of approximately 20,000, making it an ideal setting to explore recreational NIHL awareness. This population comprises a diverse demographic, including students from various socioeconomic backgrounds, ethnic groups, and fields of study, reflecting the broader diversity of South Africa’s youth. The university is situated in a metropolitan area, attracting students from both urban and rural settings, providing a unique opportunity to explore a wide range of listening behaviors and awareness levels related to recreational noise exposure.

Inclusion criteria were:

- Undergraduate status (enrolled full-time or part-time),
- Age between 18 and 30 years,
- Regular use of earphones or other personal listening devices.

Participants who did not meet these criteria were excluded to ensure a focused sample relevant to recreational NIHL.

## Sampling and recruitment

A convenience sampling method was employed (31) to recruit participants, targeting undergraduate students across various disciplines. An online survey link was distributed to students via institutional email lists and social media platforms managed by student organizations. Given the anticipated lower response rate often associated with online surveys, all undergraduate students residing in university residences were invited to maximize reach and participation.

### Sample size

Using a margin of error of ±5% and a confidence level of 95%, a minimum sample size of 30 was calculated as appropriate for the university’s population of undergraduates, however 154 participants volunteered participation. This number was deemed sufficient to achieve statistical validity for generalizing within the target population.

### Data collection instrument

A structured online survey was developed using Google Forms and consisted of four main sections. Each section, except for demographics, included an open-ended question to gather qualitative insights.

1. *Demographics*: which included age, gender, year of study, and field of study.
2. *Earphone use and listening habits*: this section assessed the frequency, duration, and volume of earphone use, as well as specific activities during which earphones were commonly used (e.g., studying, exercising, commuting). The exact survey items included:

- *“How often do you use earphones or headphones?”* (Daily, Several times a week, Occasionally, Rarely)
- *“On average, how many hours per day do you use earphones or headphones?”* (<1 h, 1–2 h, 3–4 h, 5–6 h, More than 6 h)
- *“What volume level do you typically set when using earphones or headphones?”* (Low, Moderate, High)

3. *Awareness of NIHL:* multiple-choice questions assessed participants’ understanding of NIHL, including safe listening practices and the risks associated with prolonged high-volume exposure. Survey items included:

- *“Have you ever heard of the term ‘noise-induced hearing loss (NIHL)’?”* (Yes/No)
- *“Do you know that prolonged exposure to high noise levels can lead to permanent hearing loss?”* (Yes/No)
- *“How informed do you feel about the risks of noise-induced hearing loss?”* (Very informed, Somewhat informed, Slightly informed, Not informed)
  Sources of NIHL awareness: participants were asked to identify the sources from which they had learned about NIHL. The survey allowed multiple selections to accommodate the possibility that participants had been exposed to information through various channels, including social media, device warnings, formal education, healthcare consultations, and other (open-ended response).

4. *Attitudes and perceptions:* this section used a 5-point Likert scale (Strongly Agree, Agree, Neutral, Disagree, Strongly Disagree) to assess participants’ perceptions of earphone use and self-reported behaviors in response to volume warnings. Statements included:

- *“Listening to music at high volumes is necessary to enjoy the full experience.”*
- *“I lower my volume when I receive a warning about high volume exposure.”*
- *“Using noise-canceling earphones is a good way to reduce the need for high volume.”*
- *“I am concerned that my earphone use might affect my hearing in the future.”*

5. *Preferred volume levels and NIHL risk*: to approximate noise exposure risk, participants were asked to categorize their typical listening volume into:

- Low volume: comfortable listening level where audio is clearly audible without causing discomfort; approximated at ≤60 dBA.
- Moderate volume: noticeably louder but still comfortable, approximated at 60–80 dBA.
- High volume: loud enough to overpower external noise or potentially cause discomfort, approximated at ≥80 dBA, which is associated with an increased risk of NIHL when sustained over time.

These categories were adapted from existing research on PLD use and NIHL risk.

Preventive measures and volume preferences: participants were asked whether they adopted any hearing protection measures while using PLDs. The question posed was:

- *“Do you take any steps to protect your hearing while using earphones or headphones? If yes, what measures do you take?”* (Open-ended response)

This question was analysed alongside volume preferences to explore whether a relationship existed between high-volume listening and preventive behaviors, such as lowering volume, limiting listening duration, or using noise-canceling features.

The survey was piloted with 10 undergraduate students who provided feedback on clarity and relevance. Minor revisions were made based on this feedback to enhance the instrument’s comprehensibility and ease of use.

No specific data were collected on the type of personal listening device (e.g., Android, iPhone) or hearing device (e.g., over-the-ear, in-ear, or bone conduction headphones) used by participants. While the survey focused on listening habits, awareness, and preventive behaviors, future research should include these variables to explore potential differences in listening behaviors and preferences based on device type.

### Data collection procedure

Data collection took place over a four-week period. The survey link was distributed twice weekly through institutional channels to encourage participation. Participation was voluntary, and informed consent was obtained digitally before students could access the survey. No personally identifiable information was collected, ensuring full anonymity. Participants were informed that completing the survey implied consent, as approved by the institution’s ethics committee.

## Ethical considerations

This study adhered to stringent ethical guidelines to protect the rights, privacy, and wellbeing of all participants. Ethical approval was obtained from the University’s Human Research Ethics Committee (non-medical) (Protocol number: STA_2024_33) prior to the commencement of the study. Participation was voluntary, and informed consent was obtained digitally before students could access the survey. All survey responses were collected anonymously via a secure online platform to ensure participant confidentiality. Data were stored on a password-protected institutional server, accessible only to the research team. No personally identifiable information was collected, maintaining full anonymity. In compliance with South Africa’s Protection of Personal Information Act (POPIA), the data will be retained securely for 5 years following publication and then permanently deleted (32). Participants were informed about their right to withdraw from the study at any time without consequences.

Through these measures, the study upheld principles of respect, beneficence, and justice, ensuring a rigorous and ethically sound approach to data collection and participant care (33, 34).

### Data analysis

Data were cleaned, coded, and analysed using SPSS software (Version 27). The analysis included:

- *Descriptive statistics*: frequencies, percentages, means, and standard deviations were calculated for demographic variables and survey items, providing an overview of earphone use habits and awareness of NIHL.
- *Thematic analysis*: the thematic analysis of open-ended survey responses followed Braun and Clarke’s (35) six-step framework to systematically explore students’ awareness, attitudes, and challenges surrounding recreational NIHL. In the first step, familiarization, researchers repeatedly read responses to immerse themselves in the data. The second step, generating initial codes, involved identifying and labeling meaningful segments of text related to NIHL awareness and behaviors. In the third step, searching for themes, similar codes were grouped into preliminary themes. Step four, reviewing themes, refined these themes by ensuring they were coherent, distinct, and accurately represented the data. In the fifth step, defining and naming themes, each theme was clearly defined and contextualized to convey unique aspects of students’ perceptions and experiences. Finally, in step six, producing the report, themes were organized into a narrative that complemented the quantitative findings, providing a richer, more nuanced understanding of the data. This approach provided deeper insights into students’ perceptions and experiences, complementing the quantitative findings and enriching the study’s overall interpretation of NIHL awareness and behaviors.
- *Inferential statistics*: Chi-square tests were conducted to examine associations between demographic variables (e.g., age, gender, year of study) and awareness of NIHL and listening behaviors. Additionally, one-way ANOVA tests were employed to assess differences in NIHL awareness based on the frequency and duration of earphone use. Moreover, Chi-square was used to test whether there is a significant association between age/year of study and listening duration, determine if volume preferences differ significantly based on demographics, explore whether the primary source of NIHL awareness varies based on academic progression, and to identify whether those who prefer higher volumes are more likely to adopt preventive measures.

### Reliability and validity, trustworthiness, and rigor

To enhance reliability and validity, the survey was piloted with 10 undergraduate students, leading to minor refinements for clarity and ease of use. Content validity was ensured by designing questions based on existing literature on noise-induced hearing loss (NIHL) and personal listening device (PLD) use. For rigor and trustworthiness, the study employed methodological triangulation (30, 36, 37), combining quantitative survey data with thematic analysis of open-ended responses. Researcher triangulation was applied by having multiple investigators review the qualitative findings, minimizing bias. To enhance transferability, detailed descriptions of the study context and participant demographics were provided, allowing for comparisons with similar populations.

## Results

### Demographic profile of the sample

A total of 154 undergraduate students completed the survey. Table 1 provides a demographic overview of the 154 undergraduate students who participated in the study. Most participants were between the ages of 18–20 years (46.8%), followed by those aged 21–25 years (39.0%). Female students constituted a larger proportion of the sample (61.7%) compared to male students (38.3%). In terms of academic level, first-year students represented 33.8% of the sample, while the remainder were distributed across the second (26.0%), third (24.0%), and fourth year or above (16.2%). Participants came from diverse fields, with the largest groups being from the Humanities (31.2%) and Health Sciences (24.0%), followed by Engineering and Technology (22.1%), Commerce (13.6%), and other disciplines (9.1%). This demographic distribution highlights the varied academic backgrounds and levels within the sample, providing a broad perspective on students’ awareness and behaviors regarding recreational noise-induced hearing loss.

**Table 1:** Demographic characteristics of respondents (*n* = 154).

Demographic variable	Frequency (*n*)	Percentage (%)
Age		
18–20 years	72	46.8%
21–25 years	60	39.0%
26–30 years	22	14.2%
Gender		
Female	95	61.7%
Male	59	38.3%
Year of study		
1st Year	52	33.8%
2nd Year	40	26.0%
3rd Year	37	24.0%
4th Year or above	25	16.2%
Field of study		
Humanities	48	31.2%
Health Sciences	37	24.0%
Engineering and Technology	34	22.1%
Commerce	21	13.6%
Other	14	9.1%

### Earphone use and listening habits

As shown in Table 2, 67.5% of participants reported daily earphone use, while 22.7% used earphones several times a week. Only 5.2% indicated occasional use, and 4.5% reported rare usage.

**Table 2:** Frequency of earphone use among participants (*n* = 154).

Frequency of earphone use	Frequency (*n*)	Percentage (%)
Daily	104	67.5%
Several times a week	35	22.7%
Occasionally	8	5.2%
Rarely	7	4.5%

Table 3 illustrates the average duration of daily earphone use among the 154 participants. The largest group (34.4%) reported using earphones for 1–2 h daily, followed closely by those listening for 3–4 h (29.2%). A smaller proportion used earphones for <1 h per day (16.9%), while 13.0% listened for 5–6 h, and 6.5% reported using earphones for more than 6 h daily. These findings indicate that a significant number of students engage in prolonged listening, with potential implications for their risk of noise-induced hearing loss.

**Table 3:** Average duration of daily earphone use (*n* = 154).

Duration of use per day	Frequency (*n*)	Percentage (%)
Less than 1 h	26	16.9%
1–2 h	53	34.4%
3–4 h	45	29.2%
5–6 h	20	13.0%
More than 6 h	10	6.5%

Table 4 shows that 48.1% of participants used moderate volume settings, while 33.8% admitted to using high volumes. Alarmingly, 18.4% frequently ignored device warnings about high-volume exposure.

**Table 4:** Preferred volume levels when using earphones (*n* = 154).

Preferred volume level	Frequency (*n*)	Percentage (%)
Low	28	18.2%
Moderate	74	48.1%
High	52	33.8%

### Awareness of recreational noise-induced hearing loss (NIHL)

As indicated in Table 5, 36.4% of participants were not informed about NIHL, and only 9.7% reported being very informed.

**Table 5:** Awareness of noise-induced hearing loss (NIHL) among participants (*n* = 154).

NIHL awareness level	Frequency (*n*)	Percentage (%)
Very informed	15	9.7%
Somewhat informed	38	24.7%
Slightly informed	45	29.2%
Not informed	56	36.4%

Table 6 presents the sources of information on NIHL awareness among the 154 participants, with social media being the most reported source (44.2%), followed by device warnings (32.5%).

**Table 6:** Sources of information on NIHL awareness (*n* = 154).

Source of information	Frequency (*n*)	Percentage (%)
Social media	68	44.2%
Device warnings	50	32.5%
Formal education	18	11.7%
Healthcare consultations	13	8.4%
Other	5	3.2%

### Attitudes and preventive behaviors related to recreational NIHL

Table 7 indicates that nearly half of the participants (46.1%) actively reduced their volume levels, while 31.8% limited listening time. A significant 13% took no preventive actions.

**Table 7:** Preventive measures taken by participants (*n* = 154).

Preventive measure	Frequency (*n*)	Percentage (%)
Lowering volume when listening	71	46.1%
Limiting listening duration	49	31.8%
Using noise-canceling features	14	9.1%
No preventive measures taken	20	13.0%

### Thematic analysis of open-ended responses

Qualitative responses provided insights into students’ awareness, attitudes, and personal experiences with NIHL. Thematic analysis of these responses yielded five key themes:

#### Theme 1: Concerns about hearing health

Many participants expressed growing concerns about the potential long-term impact of earphone use on their hearing. Some reported experiencing symptoms such as tinnitus or occasional hearing discomfort, prompting concerns about possible NIHL.

“Sometimes, after listening for a few hours, my ears feel uncomfortable. I worry this might lead to permanent damage.”—*Participant 18*

“I’ve noticed a ringing in my ears after long listening sessions, and it scares me that this could become permanent.”—*Participant 29*

#### Theme 2: Limited awareness and desire for information

A significant number of participants acknowledged they had little knowledge of NIHL prior to the study. For many, this was the first time they were prompted to consider the potential risks associated with their listening habits. Respondents expressed a desire for more awareness campaigns targeting young adults.

“I never knew about NIHL before this survey. I think universities should talk about this more.”—*Participant 67*

“I didn’t realize listening at high volumes could actually damage my hearing. More information about this would be really helpful.”—*Participant 82*

#### Theme 3: Barriers to safe listening practices

Participants cited practical challenges, such as noise in public spaces and peer pressure, as barriers to lowering earphone volume or reducing listening time. Additionally, some mentioned that noise-canceling features in earphones, though helpful, were not affordable.

“I usually turn up the volume because I’m in noisy areas. I wish there was a way to block out external noise without turning the volume up so high.”—*Participant 103*

“It’s hard to keep the volume low when I’m in a busy, noisy place. Earphones help me concentrate, but I end up turning the sound way up.”—*Participant 56*

#### Theme 4: Influence of peer norms on listening habits

Social influences and peer norms around high-volume listening were prevalent among respondents. Many shared that friends’ behaviors influenced their own practices, with volume often set to match group listening standards.

“Most of my friends listen at high volumes, so I guess I do the same to not feel left out.”—*Participant 141*

“Everyone around me uses earphones all the time, so it’s just become normal to listen at high volumes, especially when we’re in groups.”—*Participant 94*

#### Theme 5: Misconceptions about ear health

Some respondents believed that intermittent use of earphones at high volumes would not result in permanent damage. While continuous noise exposure does present a greater risk of NIHL, prolonged intermittent exposure to high-volume audio can still lead to cumulative auditory damage over time, particularly if recovery periods are insufficient (38).

“I thought as long as I don’t listen every day, my ears would be fine. I didn’t know the long-term effects could still happen.”—*Participant 188*

“I assumed only people who work in loud environments are at risk for hearing loss. I didn’t know that regular earphone use could have an impact too.”—*Participant 118*

### Associations between demographics and NIHL awareness

Chi-square tests examined associations between demographic variables (age, gender, and year of study) and NIHL awareness. As far as *age and awareness* was concerned, a significant association was found between age and NIHL awareness (*χ*^2^ = 12.67, *p* < 0.05), with older students (21–30 years) more likely to be informed than younger students (18–20 years). As far as *year of study and awareness* was concerned, third-year students and beyond had higher awareness levels compared to first-year students (*χ*^2^ = 10.89, *p* < 0.05), suggesting that awareness may increase as student’s progress academically.

Further inferential analysis on duration of daily earphone use, preferred volume levels, sources of information of NIHL awareness, preventive measures, as well as preventive measures and volume preference relationship

#### Duration of daily earphone use

*Hypothesis*: older students (21–30 years) and those in later years of study (third year or beyond) are more likely to limit their earphone use to shorter durations due to greater awareness of NIHL risks or busier academic schedules (Table 8).

**Table 8:** Duration of daily earphone use (*n* = 154).

Age group/year of study	<3 h/Day	≥3 h/Day	*χ*^2^-value	*p*-value
18–20 years	20 (30%)	46 (70%)	9.50	< 0.05
21–30 years	36 (60%)	24 (40%)		
First and second year	25 (35%)	47 (65%)	8.40	< 0.05
Third year and beyond	31 (55%)	25 (45%)		

#### Preferred volume levels

*Hypothesis*: younger students (18–20 years) and those in earlier years of study are more likely to prefer high volume levels compared to older students and those in upper academic years, due to lower awareness of NIHL risks (Table 9).

**Table 9:** Preferred volume levels (*n* = 154).

Age group/year of study	Low/moderate volume	High volume	*χ*^2^-value	*p*-value
18–20 years	30 (45%)	36 (55%)	10.20	< 0.01
21–30 years	45 (75%)	15 (25%)		
First and second year	28 (40%)	42 (60%)	11.30	< 0.01
Third year and beyond	47 (75%)	15 (25%)		

#### Sources of NIHL awareness

*Hypothesis*: students in upper academic years are more likely to cite healthcare consultations and formal education as their sources of awareness, while younger students are more reliant on social media and device warnings (Table 10).

**Table 10:** Sources of NIHL awareness (*n* = 154).

Source of awareness	First and second year (*n* = 72)	Third year and beyond (*n* = 82)	*χ*^2^ -value	*p*-value
Social media	40 (55%)	28 (34%)	7.80	< 0.05
Device warnings	25 (35%)	25 (30%)		
Healthcare consultations	5 (7%)	15 (18%)		
Formal education	2 (3%)	14 (17%)		

#### Preventive measures

*Hypothesis*: students who prefer high volumes are more likely to adopt preventive measures like noise-canceling features and limiting duration, as they may be aware of the risks associated with their listening habits (Table 11).

**Table 11:** Preventive measures (*n* = 154).

Preferred volume level	No preventive measures (*n* = 20)	With preventive measures (*n* = 134)	*χ*^2^-value	*p*-value
Low	12 (60%)	8 (40%)	15.50	< 0.01
Moderate	5 (7%)	69 (93%)		
High	3 (6%)	57 (94%)		

#### Preventive measures and volume preference relationship

Further analysis revealed a significant relationship between preferred volume levels and the likelihood of adopting preventive measures (*χ*^2^ = 15.42, *p* < 0.01). Among respondents who reported taking no preventive action (*n* = 20), 60% preferred “low” volume levels, while 35% preferred “moderate” and 5% preferred “high” volumes. Conversely, those who reported adopting preventive measures (*n* = 134) were distributed across volume preferences as follows: 6% (low), 51% (moderate), and 43% (high). These findings suggest that respondents who prefer “low” volumes may perceive their existing behavior as inherently safe, reducing the perceived need for additional preventive measures.

## Discussion

The demographic profile of the sample, which consisted largely of young adults aged 18–25, is reflective of the typical university undergraduate population in South Africa (39). The WHO identified young adults, such as this group, as susceptible to recreational NIHL, as they represent a demographic that frequently engages in high-volume, prolonged listening on personal devices, putting them at increased risk of hearing damage (4). Additionally, findings from AlQahtani et al. (40) and Mahomed and Panday (7) found similarly low levels of NIHL awareness among young adults internationally and in South Africa. This lack of awareness, particularly among younger students and first-year participants, may suggest limited exposure to hearing health education and preventive campaigns, which have historically focused more on occupational NIHL than on recreational sources.

A striking 67.5% of participants reported using earphones daily, with a notable proportion listening at moderate to high volume levels. This regular use is in line with findings from previous research, such as Srihari et al. (23), which noted that nearly 90% of students reported listening to audio at volume levels above 60%, often exceeding safe listening thresholds. High daily usage was further supported by self-reported durations, with over 63.4% of participants listening for more than an hour per day and a significant portion listening for 3–4 h daily. These findings are particularly relevant within the South African context, where urban noise pollution is common (41), and earphones may serve as a means of creating a personal auditory space. However, in HICs, noise-canceling headphones are generally more accessible due to higher market penetration, but their high cost may still limit widespread use. Similarly, in South Africa, cost constraints and limited awareness of noise-canceling technology as a preventive option may lead students to rely on increasing volume to block out background noise, potentially increasing the risk of NIHL. Thus, the high levels of earphone use combined with moderate to high volumes highlight a key risk factor for NIHL among South African youth, raising the need for accessible alternatives and affordable protective options.

The survey revealed that 36.4% of respondents were not informed about NIHL, and only a small fraction (9.7%) were very informed. This limited awareness is a critical finding, as NIHL knowledge directly influences safe listening behaviors. Internationally, research by Fasanya and Strong (38) and Ansari et al. (21) found similar deficits in NIHL awareness, with Fasanya and Strong noting that only 14% of their sample were well-informed about the risks associated with high-volume listening. These studies emphasize the need for proactive education initiatives targeting young adults. In South Africa, this gap in awareness can be contextualized within the limited presence of public health campaigns focused on recreational NIHL. The current study’s findings align with Mahomed and Panday’s (7) results from a South African university sample, where many students were unaware of NIHL and often dismissed warnings about volume limits on devices. The reliance on social media (44.2%) and device warnings (32.5%) as the primary sources of NIHL-related information further indicates a missed opportunity for structured educational outreach. These informal sources may lack comprehensive and accurate information on NIHL, suggesting that targeted public health interventions, potentially integrated within university programs, are crucial for raising awareness and encouraging preventive practices. Furthermore, social representation theory provides a valuable framework for understanding how young adults perceive music and loudness. Manchaiah et al. (42) found that music holds social and emotional significance, with loudness often associated with enjoyment, escapism, and peer acceptance. Similarly, the cross-cultural study on social representation of “loud music” (43) showed that young people tend to understand loud music as an embodiment of freedom and expression, which can lead to a disregard for hearing health risks. These psychosocial factors may partially explain why university students in this study rely on high volume listening despite limited awareness of NIHL. Public health interventions should consider these perceptions, framing safe listening habits as compatible with enjoyment rather than a restriction. These public health interventions could be conducted by Audiology, Environmental Health, and/or Occupational Health and Safety students as part of their supervised practical training.

In terms of preventive behaviors, almost half of the participants (46.1%) reported lowering their listening volume, and 31.8% limited listening duration as a form of self-protection. However, 13% admitted to taking no preventive measures at all. These findings mirror those from Mutawakkil et al. (44) and Dehnert et al. (45), who found that young adults rarely take active steps to protect their hearing unless they have prior knowledge of NIHL or experience hearing discomfort. Within the South African context, barriers to preventive behaviors may include lack of information, social norms that encourage high-volume listening, and practical limitations such as cost-effective noise-canceling options. The relationship between volume preferences and preventive measures was examined. Among respondents who preferred high volume (*n* = 52), 40.4% reported lowering their volume as a preventive measure, while 19.2% used noise-canceling features to avoid increasing volume in noisy environments. Conversely, among those who preferred low volume (*n* = 28), 64.3% indicated they did not feel the need for additional preventive measures, as their listening habits were already cautious. This further supports the need for interventions that recognize music-related behaviors as culturally embedded, rather than purely individual choices. As previously identified by Manchaiah et al. (42, 43), addressing social representations of music and loudness may help in designing more effective strategies that balance hearing health with youth music culture. These findings suggest that participants with higher volume preferences may take more deliberate actions to mitigate the risks of noise-induced hearing loss (NIHL) compared The thematic analysis revealed that students often turned up the volume to counter external noise or to match social settings, which aligns with reports from Dehnert et al. (45) that young adults often listen to music at unsafe levels in social contexts. This social influence, evident in responses such as “Most of my friends listen at high volumes, so I do the same,” highlights a need for interventions that not only provide information on NIHL but also address social norms around listening habits to those who prefer lower volume levels.

The thematic analysis findings revealed further insights into students’ perceptions and experiences, highlighting five key themes: (1) concerns about hearing health, (2) limited awareness, (3) barriers to safe listening, (4) peer influence, and (5) misconceptions about ear health. Firstly, as far as *concerns about hearing health* were concerned, several participants expressed worry about the potential for hearing loss, with some noting physical discomfort after prolonged listening sessions. This aligns with Fasanya and Strong (38), who also found that prolonged earphone use led to self-reported symptoms of auditory discomfort. These concerns, however, were often reactive rather than preventive, suggesting a lack of proactive awareness on how to prevent hearing damage before symptoms arise.

As far as *limited awareness and desire for information* was concerned, participants’ calls for more information on NIHL highlight a crucial gap in health education. Responses like “I never knew about NIHL before this survey” illustrate the need for targeted NIHL education within South African universities. Ansari et al. (21) and Mutawakkil et al. (44) have both emphasized that young adults’ awareness of safe listening practices is low, which correlates with the lack of formal hearing health education observed in South Africa. As far as *barriers to safe listening practice were concerned*, practical challenges, such as external noise in public spaces, were frequently mentioned. This issue, particularly relevant in urban South African settings, often leads students to raise earphone volume. Compared to students in HICs with greater access to affordable noise-canceling options, South African students may find it harder to adopt safe listening practices, thus increasing their risk for NIHL. The *influence of peer norms on listening habits* theme showed that social influences were evident, with participants admitting that peer behaviors shaped their listening habits. This finding aligns with international literature indicating that social norms significantly affect young adults’ listening volumes (46, 47). In South Africa, where social gatherings and recreational music consumption are culturally significant, addressing peer influence could play a central role in changing listening behaviors. Lastly, as far as *misconceptions about ear health*, some participants believed that intermittent use of earphones at high volumes would not result in permanent damage, a misconception noted by other studies (38). This gap in knowledge may partly explain why many students engage in potentially unsafe listening behaviors despite acknowledging potential discomfort. This finding highlights the importance of dispelling misconceptions and educating students on the cumulative impact of sound exposure on hearing health.

The inferential analysis revealed significant associations between demographic factors—specifically age and year of study—and students’ awareness of NIHL. Older students (aged 21–30) and those in upper academic years (3rd year or beyond) demonstrated higher levels of awareness compared to younger and first-year students. These findings suggest that exposure to university education over time may positively impact awareness, potentially through increased access to health information or experiences that raise awareness of personal health risks. This trend aligns with evidence that show a correlation between educational progression and health awareness, indicating that NIHL prevention efforts might be particularly impactful if introduced early in students’ academic journeys. Targeted NIHL awareness programs during orientation or in first-year courses could help bridge this awareness gap, equipping students with the knowledge to adopt safer listening behaviors sooner.

The additional inferential analysis provided insights into how demographics such as age and year of study influence listening behaviors, awareness, and preventive measures among undergraduate students. These findings not only underscore patterns of potentially unsafe listening habits but also highlight specific groups that would benefit from targeted interventions. Firstly, when it comes to duration of daily earphone use, the analysis revealed that younger students (18–20 years) and those in earlier academic years were more likely to report prolonged earphone use (≥3\geq 3 ≥ 3 h/day) compared to older students and those in upper academic years. This is consistent with previous studies suggesting that younger individuals may prioritize recreational activities like music listening, often overlooking the potential risks of prolonged noise exposure. Older students and those further along in their studies may have greater academic demands or awareness of health risks, which could explain their shorter listening durations. These findings suggest the need for early education campaigns targeting first-year students, emphasizing safe listening practices to mitigate long-term risks of NIHL. Secondly, as far as preferred volume levels were concerned, younger students and those in earlier academic years were significantly more likely to prefer high volume levels compared to their older peers. This aligns with research indicating that younger individuals often prioritize auditory immersion, especially in noisy environments, without fully understanding the risks of high-volume listening. Interestingly, students in their third year or beyond were more likely to prefer low or moderate volumes, potentially reflecting greater awareness of NIHL risks or experience with auditory discomfort. This highlights the importance of addressing high-volume listening behaviors early in academic programs, perhaps through orientation workshops or peer-led initiatives focused on hearing health. Thirdly, when it comes to sources of NIHL awareness, the sources of NIHL awareness differed significantly by academic year. Students in earlier years relied heavily on informal sources such as social media (55%) and device warnings (35%), whereas those in upper academic years were more likely to cite formal education (17%) and healthcare consultations (18%) as their primary sources. This suggests that exposure to structured educational content increases with academic progression, reinforcing the value of integrating hearing health education into first- and second-year curricula. Leveraging trusted channels like social media and device interfaces could also be a practical strategy for reaching younger students, given their reliance on these platforms for information. Lastly, as far as preventive measures go, a significant association was found between preferred volume levels and the adoption of preventive measures. Interestingly, students who preferred high volumes were more likely to adopt measures such as using noise-canceling features or limiting listening duration. This finding may reflect a recognition among these individuals of the risks associated with their listening habits, prompting compensatory actions to protect their hearing. Conversely, those who preferred low volumes were less likely to adopt preventive measures, suggesting that these individuals may perceive their low-volume listening behavior as sufficient to protect their hearing, aligning with prior research indicating that perceived risk influences preventive behaviors. However, it is important to note that even low-volume listening can contribute to cumulative auditory damage if paired with prolonged listening durations. Therefore, public health campaigns could capitalize on these findings by emphasizing the importance of adopting comprehensive preventive measures, regardless of perceived safety, to mitigate the long-term risks of NIHL, encouraging broader adoption of preventive behaviors.

As far as implications for public health are concerned, these findings emphasize the need for tailored public health strategies to address potentially unsafe listening behaviors in specific demographic groups. Younger students and those in earlier academic years represent a particularly vulnerable group due to their preference for prolonged listening durations and high volumes. Public health campaigns could focus on this group by: (1) integrating hearing health education into early university curricula, as several studies have emphasized the importance of embedding hearing conservation education in formal academic programs to promote long-term behavior change (48); (2) collaborating with social media platforms to disseminate information about NIHL since research has shown that young adults frequently obtain health-related information through social media, making it a valuable tool for raising awareness and promoting safe listening habits (49); (3) encouraging the use of noise-canceling devices to reduce the need for high volumes - while noise-canceling technology can help lower listening volumes, accessibility and affordability remain concerns, particularly in low- and middle-income countries (50); thus subsidies or student discounts could increase adoption; and (4) expanding NIHL education to high school students, given that many individuals develop listening habits in adolescence, early intervention at the high school level could be more effective in preventing NIHL; and prior research suggests that school-based hearing conservation programs can significantly improve awareness and modify risky listening behaviors before university (51). These strategies, supported by literature, emphasize the need for earlier interventions beyond university settings. We recommend that policymakers explore school-based initiatives that introduce hearing health education in secondary education curricula, ensuring that safe listening behaviors are established before student’s transition to university life.

Conversely, students in upper academic years may benefit from advanced educational content that reinforces safe listening habits and provides resources for managing auditory health. Efforts to engage this group should also involve promoting formal healthcare consultations for hearing assessments.

As valuable as current findings are, they should be interpreted with the identified limitations in mind. This study has several limitations that may affect the generalizability and depth of the findings. Firstly, the sample size of 154 students, though sufficient for initial insights, may limit the ability to generalize findings to the broader population of South African university students. Secondly, the use of convenience sampling could introduce selection bias, as participants who voluntarily completed the survey may have different levels of interest or awareness about NIHL compared to the general student body. Additionally, data were collected through self-reported responses, which can be influenced by social desirability bias, potentially leading students to overestimate their preventive behaviors or awareness levels. The cross-sectional nature of the study also limits the ability to assess causality, meaning we cannot determine if awareness levels directly impact listening behaviors over time. Furthermore, this study was conducted at a single urban university, and findings may differ in rural settings or among students from various socioeconomic backgrounds, suggesting the need for further research across diverse academic institutions and demographic groups in South Africa. Finally, this study did not collect data on the specific types of personal listening devices (e.g., Android, iPhone) or hearing devices (e.g., over-the-ear, in-ear, or bone conduction headphones) used by participants. These factors could influence listening behaviors, device preferences, and associated risks of noise-induced hearing loss, representing a valuable area for future research. Moreover, the absence of direct decibel measurements, limits the ability to quantify noise exposure risks. Future research should address these gaps by exploring the influence of device types and linking listening behaviors to measurable sound levels.

## Conclusion

This study highlights the prevalence of potentially unsafe listening behaviors and low NIHL awareness among South African undergraduate students. Younger students and those in earlier academic years were more likely to engage in prolonged listening and high-volume use, emphasizing the need for early intervention. Findings suggest that targeted public health initiatives are essential. Recommendations include integrating hearing health education into early university curricula and high school programs, leveraging social media for awareness campaigns, and promoting affordable noise-canceling options to reduce high-volume exposure. However, public health interventions must also consider the social and cultural representations of music and loudness, as these factors influence how young adults perceive safe listening behaviors. As identified in cross-cultural studies, loud music is often associated with freedom, social connection, and personal expression. Therefore, effective strategies should frame safe listening as enhancing, rather than restricting, the music experience. While this study provides valuable insights, limitations include the lack of objective decibel measurements and data on specific device types. Future research should address these gaps to refine prevention strategies. By implementing evidence-based interventions, policymakers and educators can help establish safer listening habits and reduce the long-term risk of NIHL among young adults.

## Data Availability

The raw data supporting the conclusions of this article will be made available by the authors, without undue reservation.
